# Case Report: Shadows of disappearance: the enigma of completely regressed cutaneous melanoma revealed by lymph node metastasis

**DOI:** 10.3389/fonc.2025.1671450

**Published:** 2025-10-01

**Authors:** Li Wang, Yingxue Li, Xinyan Zhang, Lin Han, Zhangyong Xia

**Affiliations:** ^1^ Department of Pathology, Liaocheng People’s Hospital, Hospital of Liaocheng University, School of Medicine, Liaocheng University, Liaocheng, China; ^2^ Department of Pathology, the Second People’s Hospital of Liaocheng, Shandong University, Jinan, China; ^3^ Department of Pathology, Liaocheng People’s Hospital, Liaocheng, China; ^4^ Department of Neurology, the Second People’s Hospital of Liaocheng, Shandong University, Jinan, China

**Keywords:** melanoma, regression, lymphadenopathy, FNAC, diagnosis

## Abstract

Primary cutaneous malignant melanoma is an aggressive neoplasm with high metastatic potential. Complete spontaneous regression of the primary lesion is rare, poorly understood, and often leads to diagnostic challenges, particularly when the cutaneous origin is no longer clinically or histologically evident. We report two cases of completely regressed cutaneous melanoma that initially presented as unexplained lymphadenopathy and were diagnosed through fine needle aspiration cytology (FNAC) and pathological examination. Both patients were admitted to non-dermatological departments due to regional lymphadenopathy. FNAC of the lymph nodes revealed malignant cells with features suggestive of melanoma. In both cases, immunocytochemical staining of cell block preparations confirmed melanocytic origin, with positivity for SOX10, HMB45, and Melan-A, and negativity for markers excluding hematolymphoid and epithelial neoplasms. Notably, neither patient had a prior history of malignancy. One patient had been misdiagnosed with onychomycosis for two years, reevaluation revealed a regressed subungual lesion while the other patient described a longstanding black lesion on the fifth fingertip that spontaneously resolved without nail involvement. These cases highlight the crucial role of cytology and immunohistochemistry in the diagnosis of metastatic melanoma when the primary lesion has fully regressed. They underscore the need for a high index of suspicion, comprehensive clinical history, and close collaboration between clinicians, cytopathologists, and dermatologists. Greater awareness of this rare presentation is essential to prevent misdiagnosis and ensure timely and accurate treatment.

## Introduction

Cutaneous melanoma is a malignant neoplasm originating from melanocytes within the epidermis and the global incidence of has been rising steadily (1, 2). Clinically, complete regression of malignant melanoma displays marked heterogeneity, typically manifesting as evolving pigmentary alterations - ranging from hyperpigmented to hypopigmented macules, patches, papules, or plaques - measuring approximately 0.4 to 3.0 cm in diameter (3). These lesions often exhibit progressive enlargement, heightened friability, spontaneous hemorrhage, and eventual involution. Notably, such transformations may precede the diagnosis of metastatic melanoma by an interval ranging from 2 months to as long as 14 years (3).

Partial spontaneous histopathological regression is a relatively frequent phenomenon in primary invasive cutaneous melanoma, with reported prevalence rates ranging from 10% to 50% (4, 5). In contrast, extensive or complete regression is exceedingly rare, with an estimated incidence of approximately 0.2% (5, 6), nevertheless, this latter phenomenon remains conceptually perplexing and is likely under-recognized in clinical practice (7). In cases where the primary lesion has undergone complete regression and metastatic melanoma is subsequently confirmed in regional lymph nodes or visceral metastases, the original cutaneous focus often becomes indiscernible or lacks sufficient histopathological evidence. In certain patients who declined biopsy of the suspected primary site, the phenomenon of regression has been documented through long-term clinical follow-up and serial photographic records (3, 8, 9). The diagnosis of completely regressed cutaneous melanoma in the absence of residual melanocytic components presents a significant conceptual and diagnostic challenge.

Two patients were admitted to different departments of our hospital with unexplained lymphadenopathy. In the course of diagnostic evaluation, fine needle aspiration (FNA) was performed, and cytopathological analysis revealed features consistent with metastatic melanoma. This prompted a retrospective investigation of their dermatologic history, through which clinicians identified prior cutaneous changes indicative of complete regression, ultimately confirming the diagnosis of completely regressed primary melanoma with nodal metastasis.

## Detailed case description

Patient A, a 71-year-old male, presented with a one-month history of a progressively enlarging, painless mass in the right inguinal region. Physical examination revealed a poorly defined, mobile, non-tender mass without overlying skin changes. Ultrasonography of the groin demonstrated multiple enlarged lymph nodes, the largest measuring 4.4 cm× 3.2 cm × 2.3 cm. This dominant node exhibited hypoechoic features with ill-defined margins, internal heterogeneity, and focal cystic degeneration. In contrast, the remaining lymph nodes appeared enlarged but retained well-circumscribed borders. Patient B, a 73-year-old elderly female, presented with unexplained lymphadenopathy in the right axillary region. Ultrasonographic examination revealed multiple hypoechoic nodules within the right axilla. The largest measured 4.2 cm × 3.4 cm × 3.0 cm and demonstrated an irregular contour with heterogeneous internal echotexture.

Neither patient had a history of malignancy, immunosuppressive disorders, use of immunosuppressive agents, nor evidence of infectious diseases, including acquired immune deficiency syndrome (AIDS). To elucidate the etiology of lymphadenopathy, clinicians requested fine needle aspiration (FNA) of the affected lymph nodes. In our institution, a distinctive feature of diagnostic practice is that superficial lymph node FNA is routinely performed by experienced pathologists, who maintain a high index of suspicion for neoplastic and lymphoproliferative conditions. Both patients presented with isolated, localized lymphadenopathy without accompanying fever. In light of these findings, comprehensive medical histories were re-evaluated and detailed physical examinations were conducted.

**Figure 1:**
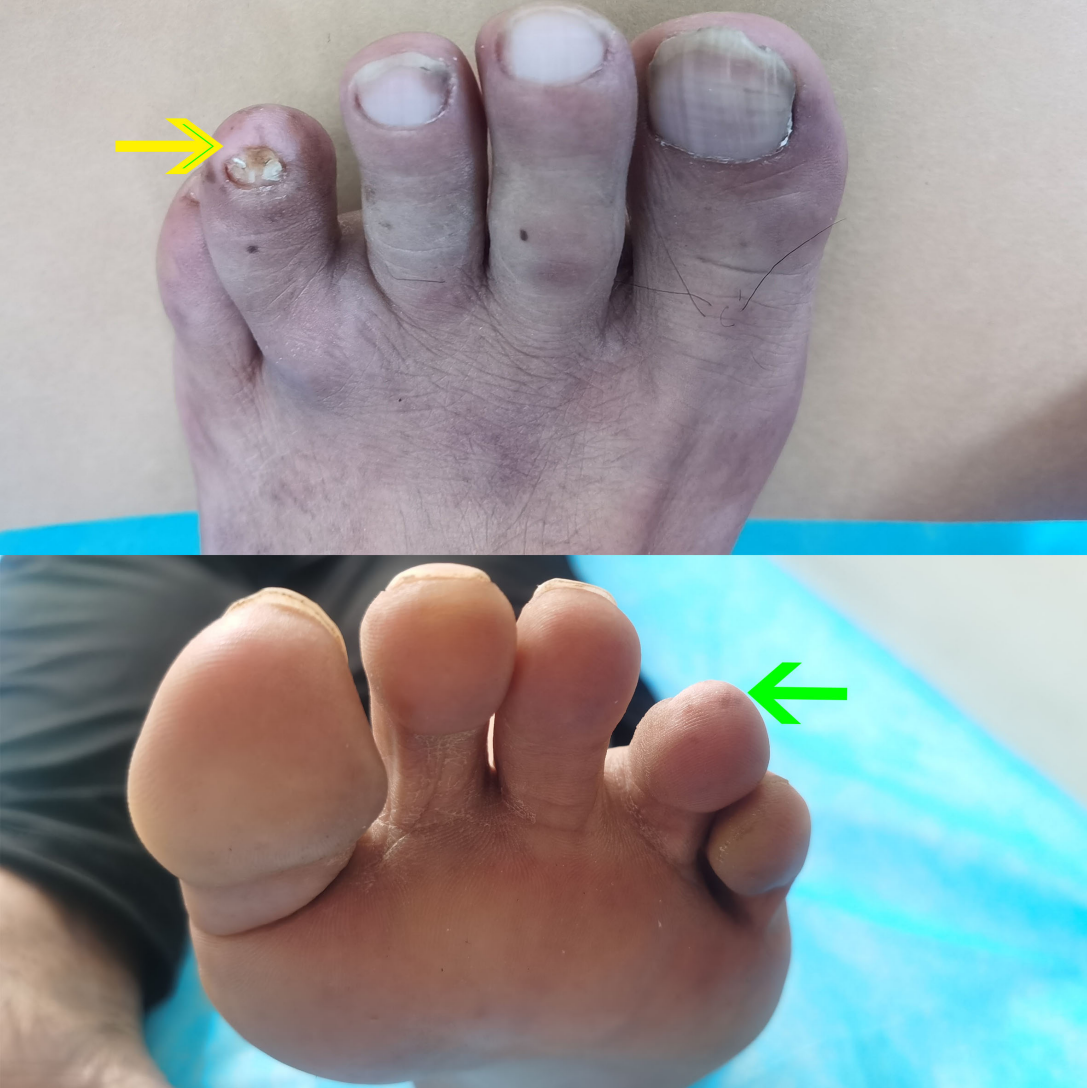
Clinical photographs of patient’s toe. A dystrophic and irregular toenail with surrounding hypopigmented skin (yellow arrow), approximately 0.8 × 0.5 cm in size, accompanied by scattered freckles in the perilesional area (green arrow), consistent with regression changes.

Patient A had been treated for onychomycosis of one toe for approximately two years, and the condition was recently considered to be in a “recovery” phase. The affected toenail appeared dystrophic and irregular, with surrounding skin exhibiting marked hypopigmentation, measuring approximately 0.8 cm × 0.5 cm while multiple scattered freckles were observed in the perilesional area (**Figure 1**). Patient B reported a long-standing history of recurrent fluid-filled lesions at the tip of the right fifth digit. Recently, the lesion underwent an abrupt and near-complete resolution. The patient recalled that the affected area previously appeared blackened, resembling a crush injury. Currently, the skin over the right fifth fingertip appears abnormally hypopigmented and regenerated in texture, resembling newly formed skin, with an area measuring approximately 0.7 cm × 0.6 cm.

**Figure 2:**
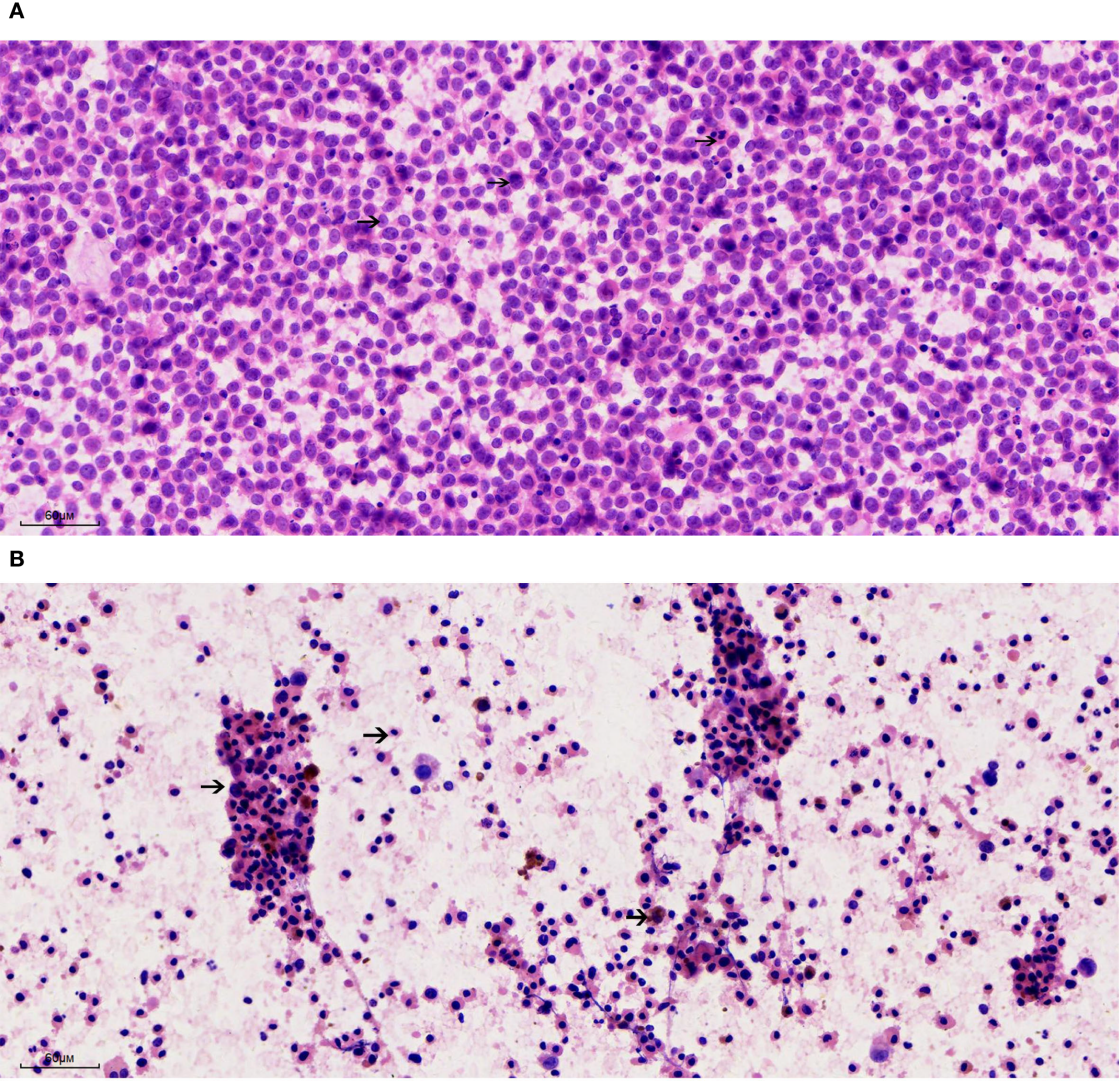
Cytological features of metastatic melanoma from lymph node fine needle aspiration (200×). **(A)** Patient A: Smear shows a dispersed population of uniform tumor cells with round nuclei, finely granular chromatin, inconspicuous nucleoli, and scant eosinophilic cytoplasm. Numerous apoptotic bodies are present; no melanin pigment is identified. **(B)** Patient B: Smear reveals a necrotic background with three distinct cell populations: (1) Malignant melanocytes with high nuclear-to-cytoplasmic ratios, coarse melanin granules, and occasional mitoses; (2) Apoptotic cells with dense, pyknotic nuclei and eosinophilic cytoplasm, some retaining melanin pigment; (3) Histiocytes with pale cytoplasm and ingested pigment.

The cytological smear obtained from patient A (**Figure 2A**) demonstrated a paucity of lymphocytic background. The tumor cells exhibited a uniform morphology and were diffusely distributed in a dispersed pattern, with minimal cellular cohesion. The nuclei were predominantly round and well-defined, characterized by finely granular chromatin and small, inconspicuous nucleoli without prominent nucleolar protrusion. Occasional mitotic figures were identified. The cytoplasm was scant and eccentrically positioned adjacent to the nucleus, displaying eosinophilic staining with heterogeneous texture; however, cytoplasmic borders were largely indistinct. Numerous apoptotic bodies were readily observed. Notably, no melanin pigment granules were detected within the specimen. In patient B (**Figure 2B**), aspiration of the right axillary lymph node yielded a small amount of dark, turbid fluid. Cytological examination of the smear revealed a markedly diminished lymphocytic component amidst a necrotic and necroinflammatory background. Cellular morphology was notably heterogeneous, with three predominant cell populations identified. A. Identified tumor cells: the malignant tumor cells were observed either as isolated single cells or loosely cohesive small clusters, characterized by a high nuclear-to-cytoplasmic ratio. These cells possessed round nuclei with finely granular chromatin and variably prominent nucleoli, which were present in a subset of the cells. The cytoplasmic volume ranged from scant to abundant and contained variable amounts of coarse melanin pigment granules. Mitotic figures, including atypical forms, were evident. B. Apoptotic cells: an abundance of apoptotic cells was noted, displaying nuclei that were markedly condensed, deeply basophilic, and exhibiting loss of recognizable chromatin architecture. Their cytoplasm appeared eosinophilic and homogeneous, with some cells retaining residual brown melanin pigment within the cytoplasm. C. Histocytes: histiocytic cells were identified, characterized by small, eccentrically placed nuclei lacking cytological atypia. These cells exhibited abundant, lightly stained cytoplasm, frequently containing phagocytosed brown melanin granules.

**Figure 3:**
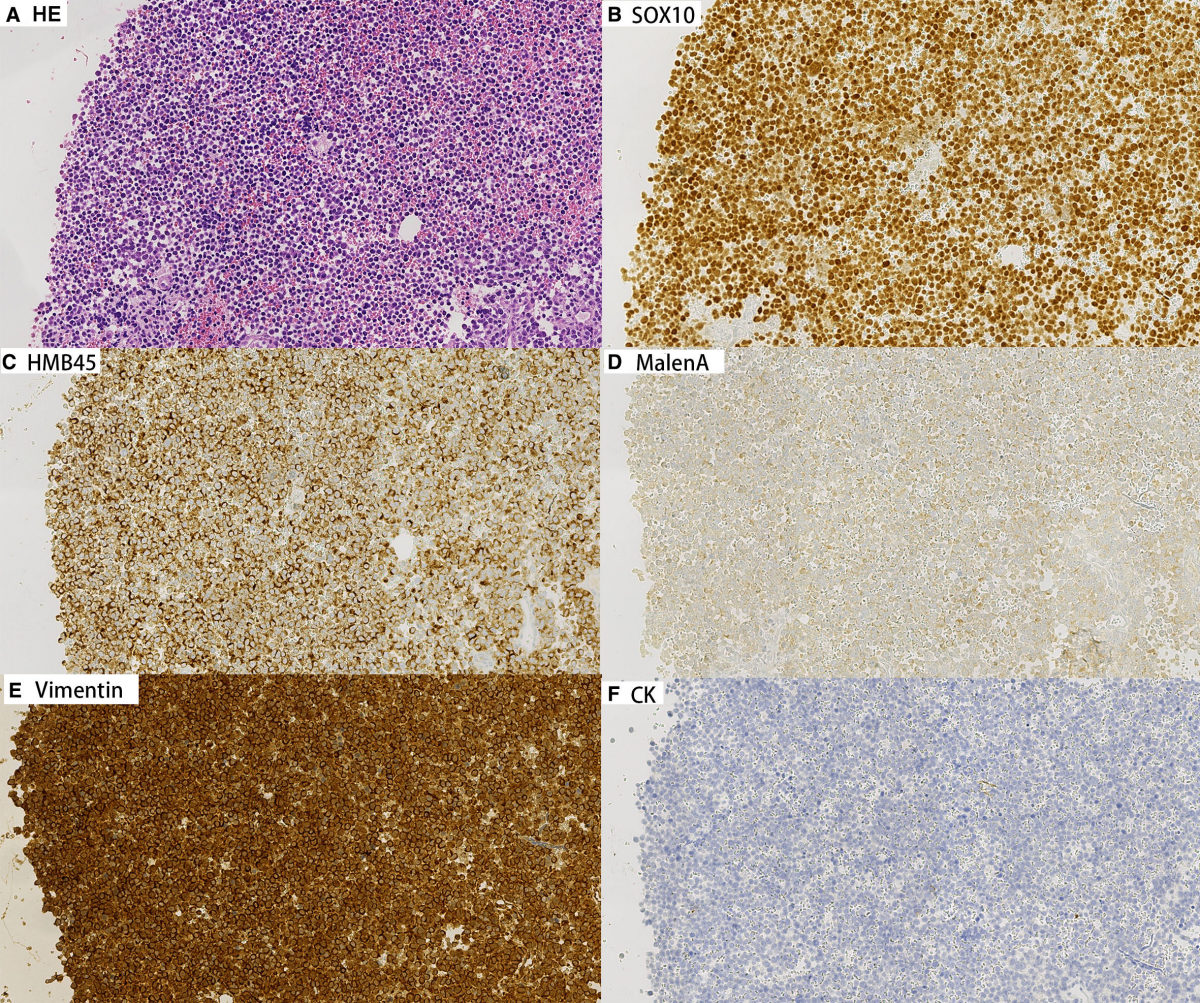
Immunohistochemical staining on cell block sections from lymph node aspirates for Patient A (200×). Tumor cells show strong positivity for melanocytic markers Vimentin, Melan-A, HMB45, and SOX10, confirming melanocytic differentiation. Negative staining for CK.

**Figure 4:**
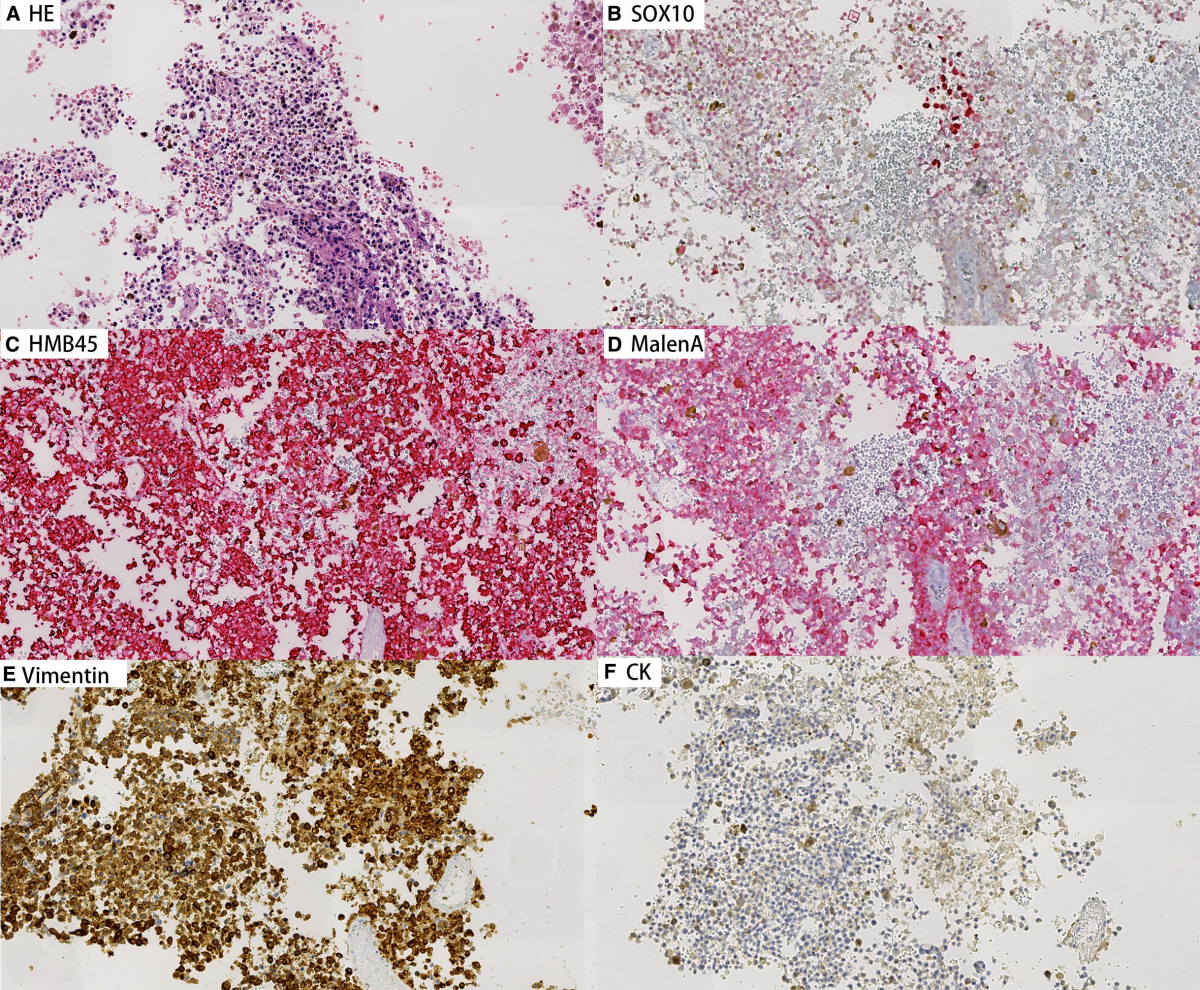
Immunohistochemical staining on cell block sections from lymph node aspirates for Patient B (200×). Tumor cells show strong positivity for melanocytic markers Vimentin, Melan-A, HMB45, and SOX10, confirming melanocytic differentiation. Negative staining for CK.

Concurrently, cell block preparations were constructed from the aspirated material, revealing morphological features consistent with those observed in the cytological smears. Immunohistochemical (IHC) analysis (**Figures 3**, **4**) demonstrated strong positivity for Vimentin, Melan-A, HMB45, and SOX10, confirming melanocytic differentiation. Conversely, markers including leukocyte common antigen (LCA), chromogranin A (CgA), synaptophysin (Syn), placental alkaline phosphatase (PLAP), and cytokeratin (CK) were negative, effectively excluding lymphoid, neuroendocrine, germ cell, and epithelial neoplasms. Molecular testing for the BRAF V600E mutation yielded wild type. Collectively, these findings established the diagnosis of metastatic malignant melanoma within the lymph nodes of both patients. In both cases, the patients had not received prior immunotherapy or other biologic treatments before the diagnosis of melanoma.

## Discussion

Spontaneous regression of cutaneous primary malignant melanoma was first reported by Smith and Stehlin in 1965 (10). In instances where complete regression of the primary cutaneous melanoma has occurred, subsequent confirmation of metastatic melanoma within regional lymph nodes and/or visceral renders the identification of the original primary lesion difficult or occasionally impossible due to insufficient residual evidence. The literature reports cases of clinically complete regression of cutaneous melanoma with nodal, cerebral, or visceral metastases, characterized by the complete absence of malignant melanocytes in the primary skin site and a marked reduction of junctional melanocytes in the overlying epidermis (3, 11). Regional lymphadenopathy is widely regarded as an essential criterion for the diagnosis of completely regressed primary cutaneous melanoma (12). In advanced stages, the presence of regression may paradoxically indicate a more aggressive tumor phenotype and adverse prognosis (13). A shared characteristic of these two cases was their protracted clinical histories marked by prior treatment, reflecting an initial lack of awareness among the attending physicians regarding cutaneous melanoma, which led to misclassification as other dermatologic conditions and missed opportunities for definitive surgical excision. In patient A, review of the symptoms and history suggests the primary lesion originated as a subungual melanoma, often manifesting as longitudinal brown or black melanonychia with onychodystrophy (14). In patient B, the lesion at the tip of the digit appeared blackened, resembling a crush injury, without nail involvement. Repeated external stimulation may have contributed to tumor progression, despite apparent clinical improvement reflecting spontaneous regression. Subsequent enlargement of regional lymph nodes prompted medical evaluation, but initial presentation to non-dermatologic specialties highlighted limitations in melanoma recognition, particularly in regressed forms.

Through careful and detailed history-taking combined with thorough physical examination, the regressive changes characteristic of cutaneous melanoma was retrospectively identified. The diagnosis of metastatic melanoma was subsequently confirmed via FNAC and immunohistochemical staining of the corresponding cell block preparations. Although neither patient underwent biopsy of the regressed cutaneous lesions, the dermatologic assessment - performed by an experienced specialist - clinically supported the diagnosis of completely regressed primary melanoma. Ultimately, the integration of cytomorphological features with comprehensive clinical information proved essential in establishing an accurate diagnosis. This case highlights the importance of interdisciplinary collaboration and the need for heightened awareness of melanoma regression among all clinicians to avoid diagnostic delays and prevent missed therapeutic windows (15).

Cytomorphologically, the aspirated black fluid from Case B provided a strong diagnostic clue suggestive of melanoma metastasis. Microscopically, brown melanin granules were readily observed within the cytoplasm of tumor cells, rendering the diagnosis of metastatic melanoma more straightforward. In contrast, Case A presented a more challenging diagnostic scenario. The tumor cells appeared morphologically homogeneous, diffusely distributed, and loosely cohesive. They exhibited scant cytoplasm, finely granular chromatin, and small basophilic nucleoli—features that are relatively nonspecific and necessitate careful differentiation from a spectrum of other neoplastic entities.

A comprehensive differential diagnosis was considered for Case A, including the following: A. Diffuse Large B-Cell Lymphoma (DLBCL): Cytologically, DLBCL is characterized by a diffuse distribution of large atypical lymphoid cells with coarse chromatin, conspicuous chromatin clumping, well-defined nuclear membranes, and a moderate amount of cytoplasm. Immunohistochemically, neoplastic cells typically express markers of the lymphoid lineage, such as LCA and CD20, while melanocytic markers including HMB45, Melan-A, and SOX10 remain negative. B. Seminoma: Fine needle aspirates of seminoma usually reveal large tumor cells arranged singly or in loose aggregates, with moderate to scant cytoplasm. The nuclei are round to slightly irregular with finely granular chromatin and one or more prominent nucleoli. A background infiltrate of lymphocytes and plasma cells is frequently observed, along with apoptotic bodies and areas of necrosis. Given that inguinal lymph nodes can be common metastatic sites for seminoma, it must be considered in the differential. IHC staining for PLAP, CD117, OCT3/4, and SALL4 supports the diagnosis of seminoma. C. Myeloid Sarcoma: Myeloid sarcomas consist of medium-sized cells with high nuclear-to-cytoplasmic ratios, scant cytoplasm, round to reniform nuclei, fine chromatin, and one or two inconspicuous nucleoli. These features may overlap with those seen in regressed melanoma, necessitating IHC analysis. The neoplastic cells typically express hematopoietic markers such as CD34, TdT, and CD99. D. Embryonal Rhabdomyosarcoma: In metastatic rhabdomyosarcoma, the cytologic smears often show small to medium-sized tumor cells with a scattered pattern. Smaller cells display narrow rims of cytoplasm, whereas larger ones show abundant eosinophilic cytoplasm with eccentric nuclei. Spindle-shaped tumor cells may also be present. Notably, intracytoplasmic cross-striations-hallmarks of rhabdomyoblastic differentiation-are typically absent in lymph node aspirates. While the morphological overlap with melanoma exists, embryonal rhabdomyosarcoma is more prevalent in pediatric populations. Diagnostic confirmation relies on positive immunostaining for Myogenin, MyoD1, and SMA.

In summary, while Case B exhibited classic features favoring metastatic melanoma, Case A required meticulous cytologic assessment and immunohistochemical profiling to distinguish it from morphologically similar malignancies. Integrating cytomorphology with clinical context and IHC results remains crucial for accurate diagnosis and appropriate therapeutic planning.

These two cases underscore significant diagnostic challenges for both clinicians and cytopathologists. For clinicians, axillary lymphadenopathy in female patients is most commonly attributed to metastatic breast carcinoma, which explains why Patient B initially sought evaluation in the breast surgery department. In contrast, inguinal lymphadenopathy is frequently caused by reactive hyperplasia secondary to local inflammation or infection, with metastatic malignancy as a less common but important differential diagnosis. In cases of inguinal lymph node metastasis without an identifiable primary tumor, clinicians should maintain a high index of suspicion for cutaneous melanoma, especially after ruling out more common primary sites. Prompt recognition of this uncommon presentation is critical to avoid diagnostic delays and to facilitate timely and appropriate management. In both of the reported cases, the limited awareness and understanding of cutaneous melanoma among non-dermatology specialists contributed to the initial oversight of the primary skin lesions. This highlights the importance of multidisciplinary collaboration and the need for heightened clinical vigilance. Notably, acral melanoma-the most prevalent melanoma subtype among East Asian populations-is often overlooked due to its atypical presentation and frequent occurrence on less-visible anatomical sites such as the palms, soles, and subungual regions. This subtype is associated with a poorer prognosis, largely owing to delays in diagnosis and a higher likelihood of advanced disease at presentation (16). In the Caucasian population, approximately 77.5% of acral melanomas are diagnosed as invasive at the time of presentation. Notably, melanomas located on the foot demonstrate an even higher proportion of invasive disease, underscoring the aggressive nature and diagnostic challenges associated with acral melanoma in these anatomical sites (17). Explanations for the phenomenon of regression in malignant melanoma have predominantly centered on immunologic mechanisms, with evidence suggesting that host immune responses-particularly cytotoxic T-cell activity-play a pivotal role in the elimination of tumor cells and subsequent regression of the primary lesion (18). Regression of the primary tumor following lymph node or visceral metastasis is thought to reflect an active immune response against melanoma cells (19, 20). Both innate and adaptive immunity contribute to this process, which arises from the dynamic interaction between tumor cells and the surrounding microenvironment. In particular, tumor-infiltrating lymphocytes target melanocytic tumor cells, leading to their destruction and replacement by fibrotic tissue (21, 22). The presence of metastatic melanoma within a regional lymph node may serve as a catalyst for a systemic immune response, potentially resulting in the immunologic targeting and subsequent regression of the primary cutaneous lesion. However, in advanced-stage melanoma, the occurrence of regression may paradoxically indicate a more aggressive biological behavior and is often associated with poorer clinical outcomes (13).

The primary cutaneous lesions in these cases were identified by cytologists with subspecialty training in dermatopathology and subsequently confirmed by dermatologists. In the absence of an identifiable primary lesion, the cytomorphological features of tumor cells are often nonspecific and necessitate careful differentiation from a range of other malignancies, including diffuse large B-cell lymphoma, seminoma, myeloid sarcoma, and embryonal rhabdomyosarcoma. Although the presence of intracellular melanin pigment can provide a valuable diagnostic clue, it is observed in no more than 50% of melanoma cases, further complicating the diagnostic process (13).

Melanoma has been aptly termed the “greatest pretender” due to its remarkable cellular heterogeneity and phenotypic plasticity, which enable it to mimic a wide spectrum of histopathological appearances and complicate accurate diagnosis (23). This report highlights the diagnostic and therapeutic challenges encountered in two patients, underscoring that complete regression of primary cutaneous melanoma frequently results in clinical and pathological misdiagnosis or oversight. The presence of lymph node metastasis in the absence of an identifiable or adequately evidenced primary lesion significantly complicates both diagnosis and management. Accordingly, heightened vigilance and awareness among clinicians and pathologists are imperative to improve recognition, avoid diagnostic pitfalls, and optimize patient outcomes in such complex cases. This process involves not only detailed dermatologic examination but also comprehensive inspection of all mucosal surfaces, including oral, nasal, anogenital, and ocular regions, where occult melanomas may arise. Multidisciplinary evaluation is essential, incorporating specialists such as gynecologists, ophthalmologists, otolaryngologists, and gastroenterologists, depending on the clinical context. Such a systematic approach ensures that occult or atypically located primary melanomas are not overlooked, allowing for accurate staging, guiding appropriate therapeutic strategies.

## Data Availability

The original contributions presented in the study are included in the article/supplementary material. Further inquiries can be directed to the corresponding author.
